# High-Throughput Analysis of *in-vitro* LFP Electrophysiological Signals: A validated workflow/software package

**DOI:** 10.1038/s41598-017-03269-9

**Published:** 2017-06-08

**Authors:** P. Tsakanikas, C. Sigalas, P. Rigas, I. Skaliora

**Affiliations:** Biomedical Research Foundation, Academy of Athens, Center of Basic Research, Athens, Greece

## Abstract

Synchronized brain activity in the form of alternating epochs of massive persistent network activity and periods of generalized neural silence, has been extensively studied as a fundamental form of circuit dynamics, important for many cognitive functions including short-term memory, memory consolidation, or attentional modulation. A key element in such studies is the accurate determination of the timing and duration of those network events. The local field potential (LFP) is a particularly attractive method for recording network activity, because it allows for long and stable recordings from multiple sites, allowing researchers to estimate the functional connectivity of local networks. Here, we present a computational method for the automatic detection and quantification of *in-vitro* LFP events, aiming to overcome the limitations of current approaches (e.g. slow analysis speed, arbitrary threshold-based detection and lack of reproducibility across and within experiments). The developed method is based on the implementation of established signal processing and machine learning approaches, is fully automated and depends solely on the data. In addition, it is fast, highly efficient and reproducible. The performance of the software is compared against semi-manual analysis and validated by verification of prior biological knowledge.

## Introduction

Electrical brain activity recordings provide important diagnostic and research tools. Brain signals vary widely in their spatiotemporal characteristics and means of acquisition. Since the development of the electroencephalogram (EEG), electrical signals have been obtained by gradually more invasive methods and at increasingly higher spatial resolution: from recording electrical activity from the top of the skull (EEG) or the surface of the cerebral cortex (i.e. electro-corticogram, ECoG), to recordings within brain tissue: local field potentials (LFPs), or action potentials from individual cells (Multi- and Single-unit activity).

Among these, field potential activity represents the slow voltage fluctuations (<200 Hz) that are caused by ionic currents in the vicinity of the recording electrode^1, 2^. LFPs have become increasingly popular over the last decade as they offer multiple insights into the mechanisms underlying cortical processing^1^. Their different band-limited components (e.g. delta, theta, alpha, beta and gamma bands) enable scientists to observe the integrative excitatory and inhibitory synaptic processes at the network level. Also LFPs carry information about the state of the cortical network, local intra-cortical processing and the effect of neuro-modulatory pathways^3, 4^. Another reason for the scientific interest in LFPs is that they typically contain a broad spectrum of neural oscillators ranging from under 1 Hz to over 100 Hz and reflecting the activity of several neural processing pathways^5–8^. Additionally, LFP signals allow the empirical examination of distinct and potentially independent information channels in neural processing^9–11^, while in combination with spike recordings, can give insight into circuit mechanisms generating neuronal activity^12^. Finally, information extracted from LFP signals is used for the development of *in silico* models of neuronal activity^13, 14^, which are then compared to *in-vivo* experiments. In systems neuroscience there is a requirement for massively parallel data from multi-electrode arrays, implying a tremendous increase of analysis load. Therefore, for all the aforementioned reasons, the need for reliable, automated and high throughput tools for detecting and quantifying neural events in LFP recordings is of critical importance.

Currently, several software packages (commercial and academic) are available for analysis of neuronal signals, such as EEGLAB^15^, CHRONUX^16^, OSort^17^, and Spike2^18^. While all those packages are very efficient in terms of time-frequency and spike-train analysis, none allows for an automatic detection and quantification of the irregular and variable voltage bursts inherent in field potential recordings. Several lab-based studies came up with diverse solutions. For instance, Volgushev *et al*.^19^ and Seamari *et al*.^20^ performed LFP event detection by combining intracellular and extracellular recordings. They used the intracellular recordings in order to detect the events and applied the detection results on the LFP signal. Other groups developed, methods for event detection based on multiunit activity^21, 22^, which, however, has different properties compared to LFPs, including higher acquisition frequencies and event detection based on neuronal spikes. Another common approach employs arbitrary user-defined global thresholds (Sanchez-Vives *et al*.^23^; Compte *et al*.^24^), any value above which is considered an event. Since those methods are not data driven and are applied globally, they can easily lead to either overestimation or underestimation of LFP events both in terms of occurrence and onset/offset values. In addition, they are highly time consuming (since they involve extensive trial and error in order to set the threshold and possibly also subsequent manual verification of the detection accuracy), and subjective since the determination of an appropriate threshold can vary significantly between studies with different or even the same experimental models.

A significant development in this direction was provided in 2007 by Mukovski and colelagues^25^ for event detection from LFP signals *in-vivo*. Their method applied an automatically estimated global threshold on a pre-processed LFP signal. The threshold is guided by defining several justified, albeit user-defined, assumptions (e.g. limit set of 50% of data sorted by amplitude). Although this approach provides a data-driven automatically defined threshold for event detection, it has certain features that limit its widespread use: (i) it requires a pre-processing step that removes the low frequency fluctuations (<20 Hz); and (ii) the estimated threshold is applied globally to the entire recording. These features could be problematic in cases where the lower frequencies are an integral part of the signal (e.g. add ref), or in recordings that exhibit dynamic baseline changes and/or events with low periodicity.

Hence, in most studies investigating activity in the LFP domain (e.g. Up states, network oscillations, K complexes, spindle bursts), the detection is either performed on signals other than LFP^19–22^, or employs global thresholds (whether arbitrary or data-derived), that are insufficient for accurate detection of the events’ onset and offset in dissimilar datasets. With this in mind, and considering the increasing interest in LFP signal exploitation, as well as the technological advances in multiple simultaneous field potential recordings (e.g. multi electrode arrays - MEAs) employed both *in vitro* and *in vivo*, we have developed a novel workflow for the automatic detection and quantification of spontaneous activity bursts in LFP recordings, employing and adapting methods from the fields of Signal Processing, Speech Analysis and Machine Learning. From the user’s point of view, our methodology (which we call LFPAnalyzer) does not require knowledge of the aforementioned scientific areas, nor prior assumptions on the recorded signal from the user. All processing and decisions are data driven, thereby allowing a direct comparison of the results obtained by different users within and across laboratories. Moreover, and in contrast to the previous attempts (e.g. Mukovski *et al*.)^25^, the presented methodology is locally adaptive, enabling a more accurate threshold estimation and thus more precise estimation of onsets and offsets. In addition, the analysis is applied to the full (unfiltered) signal, and is fully automated in terms of the detection of recurring events and the estimation of a plethora of their properties, as presented in detail in the Methods Section. Here we provide a three-fold assessment of the efficiency and robustness of the proposed workflow by: (a) comparing its accuracy and reproducibility against manual processing, (b) validating the obtained data against already published pharmacological results; and (c) comparing the efficiency of the local vs. global thresholding schemes on the same datasets. To the best of our knowledge this is the first attempt in the field of computational neuroscience towards a high throughput and fully automated software package, with local and adaptive threshold estimation based solely on the data.

## Results

### Outline of Signal processing and event detection system

**Figure 1:**
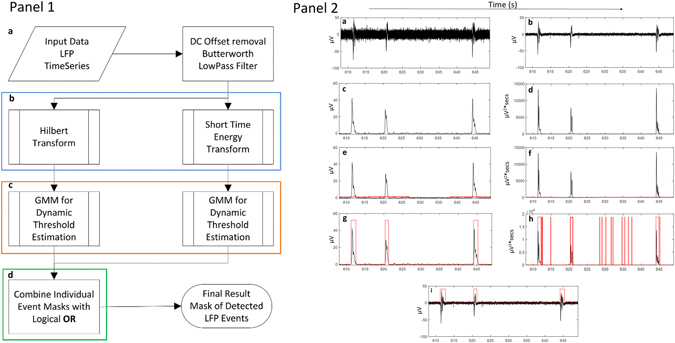
Panel 1: Workflow schematic presentation, including four distinct steps: **(a)** the input data is pre-processed (removal of DC offset and low pass filtering), **(b)** two feature sequences are extracted for each segment, based on two complementary mathematical transformations (Hilbert and Short Energy; blue rectangle); **(c)** a dynamic threshold is estimated for each feature sequence (orange rectangle), and used to create a mask; **(d)** the two masks are combined using a logical OR operator (green rectangle) and used for the detection of the onset and offset of the LFP events. Panel 2: Illustration of the successive processing of the LFP signal **(a)** Original signal; **(b)** preprocessed signal, after DC offset subtraction and low-pass filtering at 200 Hz; **(c)** Hilbert transformed signal; **(d)** short time energy transformed signal; **(e)** Hilbert transformed signal (black line) with estimated threshold levels (red line); **(f)** short time energy transformed signal (black line) with estimated threshold levels (red line); **(g)** detected events (red line) on the Hilbert signal; **(h)** detected events (red line) on the short time energy signal; **(i)** final result with the detected events (red line).

The developed workflow for automatic analysis and event detection in LFP signals, is implemented in MATLAB^©^ environment. A brief description is outlined in Fig. 1 (Panel1): (1) Signal pre-processing: DC offset is subtracted and the trace is low-pass filtered to isolate the LFP-related component (frequency range <200 Hz); (2) Computation of two complementary feature sequences, each based on a different transformation process: namely Hilbert transform^26^ and Short time energy transform^27^. Both feature sequences are used for the efficient separation of the LFP signal events from the baseline; (3) Estimation of a dynamic data-driven threshold for each feature sequence (based on Gaussian Mixture Models^28^), ensuring no *a priori* assumptions about the particular shape or absolute size of the event; and (4) post-processing the event-containing segments from each feature sequence with an OR logical function and extraction of the final detected LFP events. Please refer to Methods Section for more details.

### Evaluation of LFPAnalyzer

#### Accuracy

In order to validate the developed methodology, we first compared the results obtained by LFPAnalyzer to those obtained by manual analysis. This comparison provides a crucial indication of our method’s efficiency to deal with real data. The manual analysis consisted of the same logical steps, i.e. pre-processing (DC subtraction and low-pass filter), Hilbert transform, and global threshold setting at 40% of the standard deviation of the input signal in order to help the users identify the active states and refine the boundaries of the events. The detected events were then inspected by an experienced user so as to define their onset and offset. At this point we should state that, as with any method that employs a threshold setting, events with amplitude below the user-defined threshold value were omitted.

**Figure 2:**
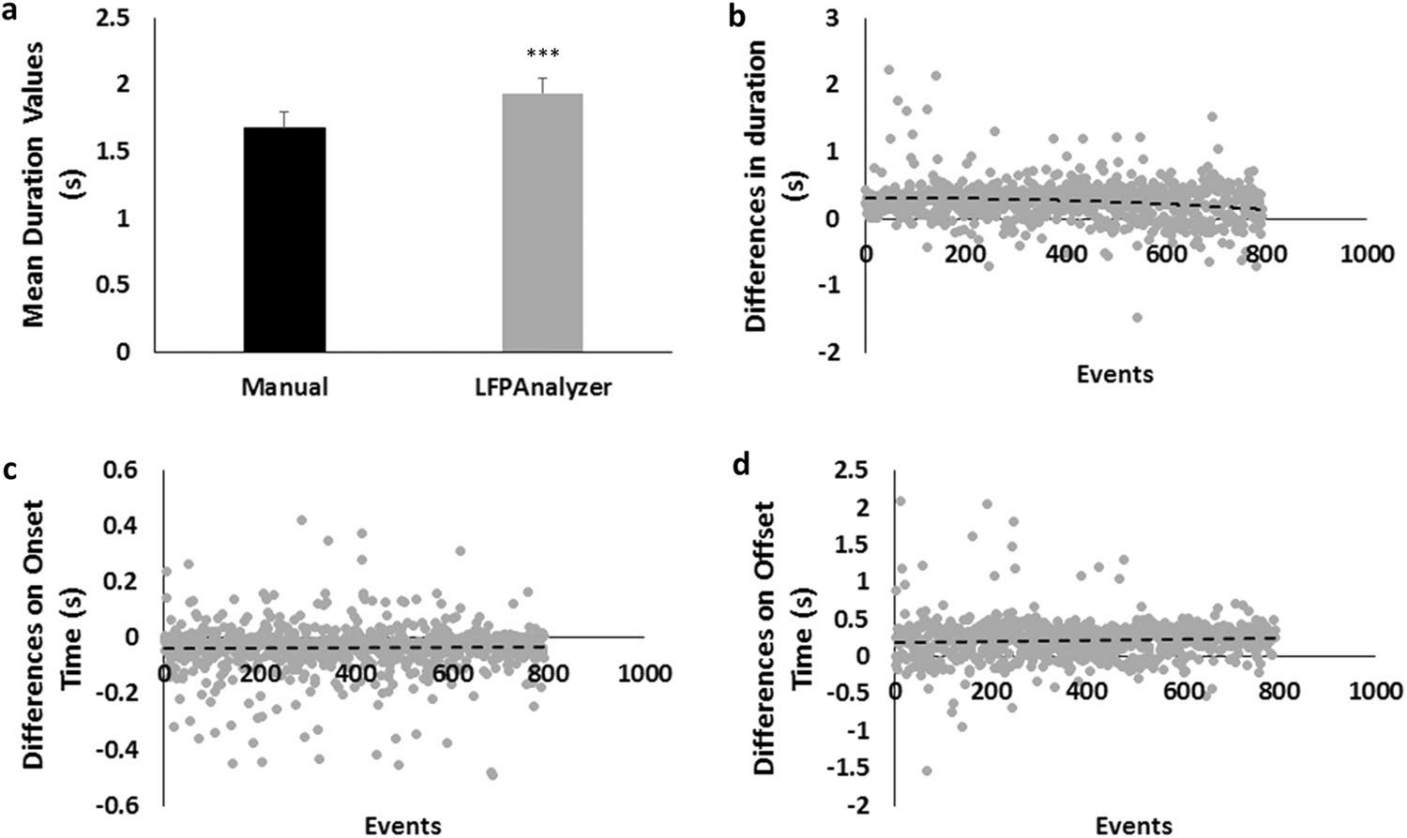
Comparison of LFPAnalyzer vs. manually extracted results. **(a)** Average duration values obtained with the manual (black bar) and the LFPAnalyzer (grey bar), error bars indicate the SEM, **(b)** Scatter-plot of the differences in individual values of event duration (LFPAnalyzer minus manual detection). **(c)** Scatter-plot of the differences in individual values of event onset (LFPAnalyzer minus manual detection). **(d)** Scatter-plot of the differences in individual values of event offset (LFPAnalyzer minus manual detection).

**Figure 3:**
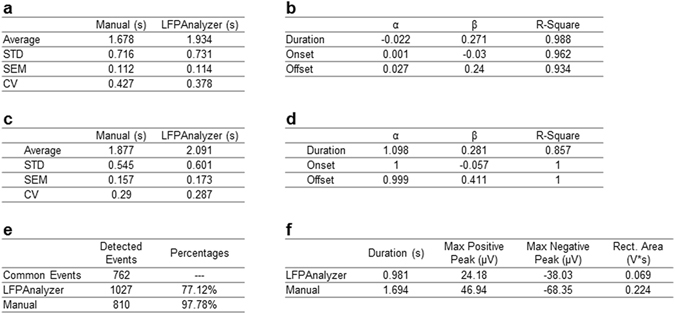
Tables’ display item. **(a)** Average values of duration, and the corresponding standard deviation, standard error of means, and coefficient of variation, for the two analysis methods. **(b)** Linear Regression parameters. Line 1 shows α, b and R-Square values for the duration, line 2 for the onset and line 3 for the offset. **(c)** Average values of duration, and the corresponding standard deviation, standard error of means, and coefficient of variation (CV) obtained with manual and automatic processing. **(d)** Linear Regression parameters. Line 1 shows α, b and R-Square values for the duration, line 2 for the onset and line 3 for the offset. **(e)** Number of events detected by LFPAnalyzer and manual approaches. **(f)** Average values of parameters for the additional events (n = 217) detected by LFPAnalyzer vs. the common events (n = 762) detected by both manual and automated methods.

Figure 2a illustrates the comparison of measurements concerning event duration. Mean values obtained with the two methods showed an average difference of ∼0.26 s (Fig. 3a), statistically significant with t-test and p ≪ 10^−7^, while the standard deviation was similar (0.73 and 0.72 for the LFPAnalyzer and the manual analysis respectively). Interestingly, the coefficient of variation (CV), which is a standardized measure of the dispersion of a probability distribution, is smaller for the LFPAnalyzer compared to the manual approach, reflecting the precision and repeatability of the analysis method. In order to further explore the source of the moderate (15%) deviation between the two methods, we calculated the differences in duration, onset and offset values for each event (Fig. 2b–d).

Figure 3b presents the values for *α* and *b*, as well as *R-square* values, showing the goodness-of-fit (e.g. *R-square* = *1*, indicates a perfect fit of the regression function to the data). As can be seen from Figs 2 and 3b, in the case of event duration the gradient value is almost zero (−0.022) with a bias of 0.271 s; in close agreement to the difference in average values (∼0.26 s). The regression lines for Onset and Offset differences are also parallel to the x-axis, and the ‘bias’ introduced by the LFPAnalyzer is −0.03 and 0.24 s for the onset and the offset, respectively. Hence we can conclude that the LFPAnalyzer produces a consistent ‘overestimation’ of event duration compared to the user, which is almost entirely due to the detection of event offset. It should be noted that the characterization of over- or under-estimation is based on the assumption that the user-defined measurements are taken as the “ground” truth. The consistent overestimation on the event offset is a systematic bias since the same properties (detection conditions) are satisfied by including the slow slope of the event at its end (when the bursts return the baseline level and rests there).

#### Reproducibility

Any automated method is by definition highly reproducible. In order to obtain a quantification of LFPAnalyzer’s advantage we examined the results obtained by two independent experts using the manual approach on the same dataset (18 recordings), and examined the onset, offset and duration of detected LFP events. A linear regression applied on the duration values, presented in Fig. 3c, revealed a good correspondence between the two users (*α* = 1.098), but an 11.4% difference in values (b = 0.281 s), a value almost equal to the average duration difference (Fig. 3d).

**Figure 4:**
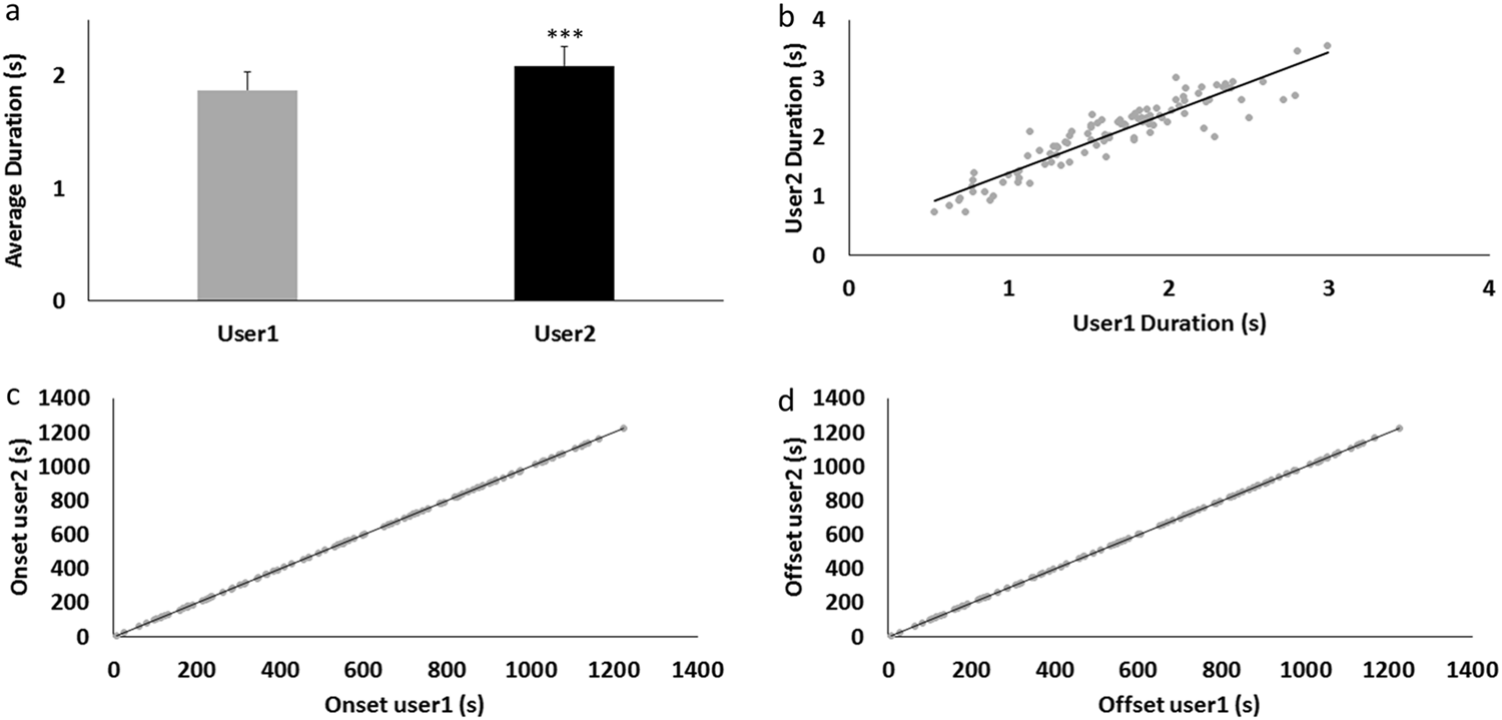
Reproducibility assessment: **(a)** average duration times on the same dataset by two users, the error bars corresponds to SEM. Average duration difference is ∼0.21 s. **(b)** Duration values for each event, and the corresponding regression line. Scatter plots of onset **(c)** and offset **(d)** values for each event, and the corresponding regression lines.

Hence, although we have a one-to-one relationship between duration values computed by each user, there is a significant discrepancy in absolute values (see Fig. 4a). This precludes the direct comparison of data analyzed by different users, since this difference (the bias) is not the same for each event (see Fig. 4b), but is instead a regression bias.

In order to further explore the basis for this difference between the two users, we performed the same analytic comparison on the onset and offset timings. Regression fits for both onset and offset values are illustrated in Fig. 4c,d and reveal a perfect one to one relationship (*α*_*onset*_ = 1 and *α*_*offset*_ = 1). Interestingly, although there is a practically non-existent bias in onset values (*b*_*onset*_ = −0.06 s), the bias for offset values was significant (*b*_*offset*_ = −0.411 s), suggesting that user1 tends to overestimate (or user2 tends to underestimate) the precise point where an event ends (Supplementary Material 1). Taken together, we can conclude that, unlike the automated LFPanalyzer presented here, the manual approach is prone to the users’ random bias. It is also noteworthy that the discrepancy in event duration between the two users is similar to the discrepancy between the two methods (11.4% vs. 15%), and both are accounted by the different estimation of event *offset*. This indicates that the termination of an LFP event is more prone to subjective assessment when performed manually, and that this subjectivity is eliminated by the automatic evaluation of the recorded events.

#### Efficiency

Finally, we examined the efficiency of the two approaches in terms of numbers of event detected. As can be seen in Fig. 3e, LFPAnalyzer successfully detects 98% of the LFP events detected by the manual analysis, indicating that practically all events detected by the users are also detected by the software. At the same time, the automated method detects 217 (∼30%) additional events. In order to locate the source of this discrepancy, we examined the parameters of these ‘extra’ 217 events in terms of average duration, max/min amplitude and rectified area (Fig. 3f). We find that the additional events are overall significantly smaller (p ≪ 10^−4^) compared to the average size of the shared detected events, in terms of duration (0.98 vs. 1.69 s); rectified area (i.e. the sum of absolute voltage values within an event) (0.069 vs. 0.224 V*s); minimum and maximum amplitude (−38 vs. −68 µV and 24 vs. 49 µV, respectively). On the other hand, the spectra power content across the five frequency bands (Delta, Theta, Alpha, Beta, and Gamma) are similar, i.e. not statistically different (unpaired t-test > 0.05 for each band), to the events shared by the two detection methods (Supplementary Material 2).

### Validation against previously published pharmacological manipulations

**Figure 5:**
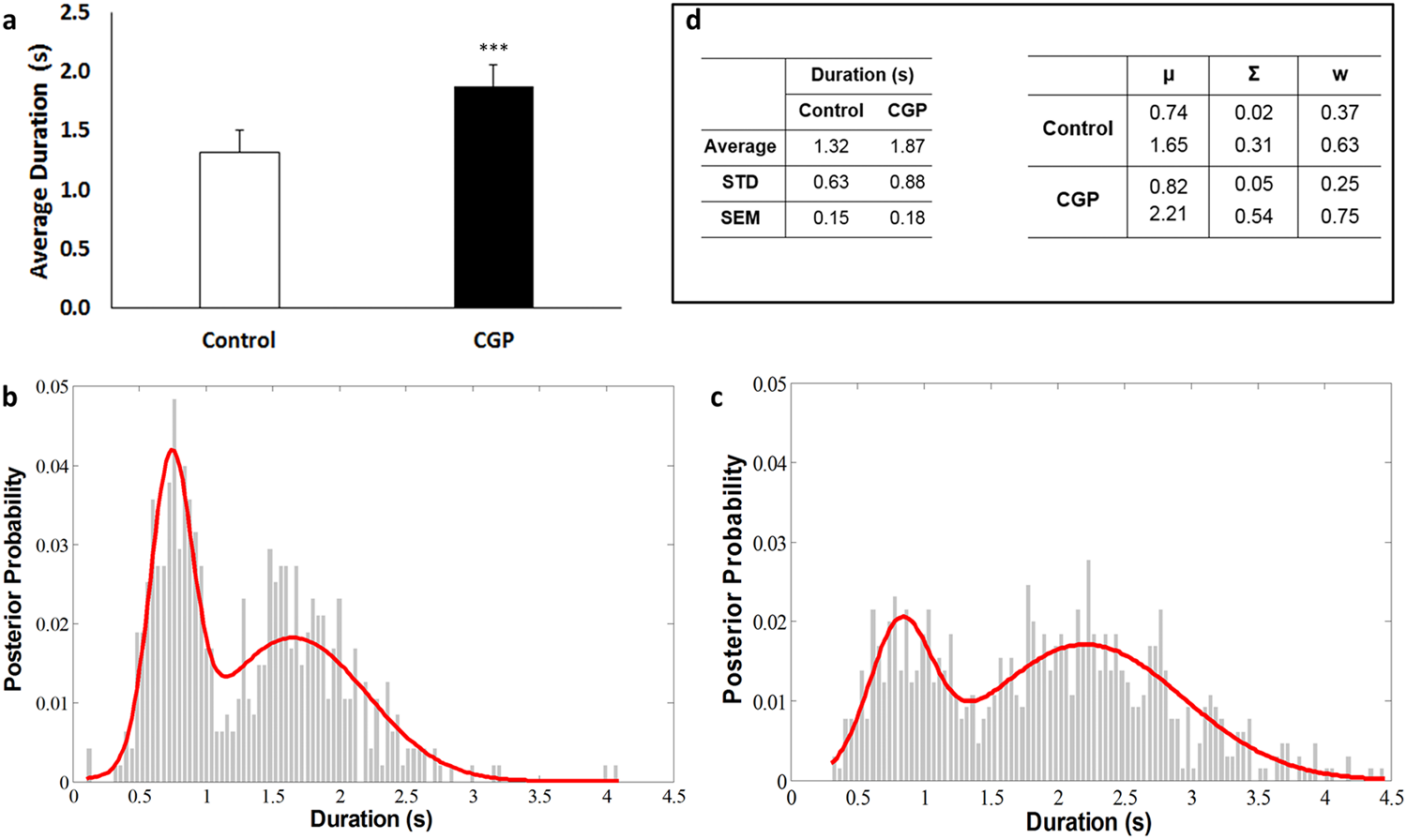
Effect of CGP (1 μΜ) on events in LFP recordings. **(a)** Event duration is significantly increased (1.32 ± 0.15 vs 1.87 ± 0.18 s, or ∼42%). Error bars indicate SEM, p < 0.001, student’s t-test. **(b)** GMMs fit on duration measurements for the control samples. **(c)** GMMs fit on duration observations for the CGP samples. **(d)** Tables showing the values presented in Fig. 5a,b and c. μ is the mean value, Σ is the covariance matrix and w the proportion of each distribution in the whole distribution model.

A further assessment of our method was to compare the results obtained from LFPAnalyzer against previously published biological findings. Up states are spontaneous epochs of persistent depolarizations that occur in a highly synchronized manner and can therefore be detected both in intracellular and in LFP recordings^29^. These network events are maintained by recurrent excitation within local circuits and can be turned on and off by synaptic input^30, 31^. Previous studies have shown that GABAergic inhibition is essential for stabilizing persistent activity by balancing excitation and terminating the Up state. Furthermore, GABA_A_ and GABA_B_ receptor-mediated inhibition have complementary roles in regulating persistent activity: blockade of GABA_B_ receptors increases^32–34^, while suppression of GABA_A_ receptors decreases^33^ the duration of spontaneous Up states. In these studies the effect of GABA_B_ receptors on Up state duration was studied with both intracellular and extracellular (LFP) recordings. Here, we relied exclusively on LFP recordings of spontaneous Up states while using the same experimental conditions. Since it is already established that the correspondence between extracellularly and intracellularly recorded Up states is very strong^34, 35^, we expected to find a similar effect of GABA_B_ receptor blockade using the developed package. Following the published experimental protocol^32, 34^, we examined spontaneous cortical events in the presence of 1 μM CGP55845^36^. Events were detected and quantified with LFPanalyzer and the results are compared to the published effect regarding their duration (Fig. 5). As illustrated in Fig. 5a, the results in both sample groups (control and CGP-treated) are fully compatible to the literature: event duration is increased from 1.32 ± 0.15 s in the control group to 1.87 ± 0.18 s in the CGP-treated group (or ∼42% increment, p < 0.01, student’s t-test), in agreement to the findings reported both by Mann *et al*.^32^, and Sigalas *et al*.^34^.

In addition, we further investigated the differences between control and CGP-treated samples, by estimating the corresponding probability density functions (pdfs). Gaussian Mixture Models^28^ applied to duration values revealed a bimodal distribution, as shown in Fig. 5b,c. Those mixture pdfs are defined as: $p(x|\lambda )={\sum }_{i=1}^{M}{w}_{i}g(x|{\mu }_{\iota },{\Sigma }_{\iota })$, where x is the 1-dimensional observations of duration, *w*_*i*_ *, i* = 1, …, M the mixture weights with ${\sum }_{i=1}^{M}{w}_{i}=1$, *g* is the Gaussian function, *μ* and *Σ* the mean and covariance, and *λ* the full set of parameters $\lambda =\{{w}_{i},{\mu }_{i},{\Sigma }_{i}\}$. Here we used a two component GMM (*M* = 2) since the corresponding distributions are clearly bimodal, and the resulting parameters are shown in Fig. 5d. This analysis indicates that each biological state (control, CGP) is best described by 2 populations of events, with different average durations. Hence, in the control state there is one population with a shorter average duration (μ_1_ = 0.74 s), which represents about a third of the sample (w_1_ = 0.37); and a second population with a longer average duration (μ_2_ = 1.65 s), which accounts for the remaining two thirds of the total sample (w_2_ = 0.63). In contrast, in the CGP state, the first population exhibits an increased mean duration (μ_1_ = 0.82 s), but now accounts for only a fourth of the total dataset compared to the corresponding control (w_1_ = 0.25); while the second population also exhibits longer mean duration (μ_2_ = 2.21 s) and also account for a greater percentage of the dataset (w_2_ = 0.75) compared to control samples. The aforementioned analysis can be applied to any of the additional properties quantified by the developed method (as mentioned in the Methods) in order to quantify and compare different aspects of LFP events.

### Anticipated Application

Finally, we present a prospect application of the developed software towards systems neuroscience since, as a high-throughput methodology, it can lead to the production of massive data via event modelling. On that basis, we estimated the probability density functions for 10 distinct event properties extracted from the control samples presented above. In addition to event duration, these include the time between two consecutives LFP events (inter-event interval), min and max values, rectified area, power in the five frequency bands: i.e. delta, theta, alpha, beta and gamma (presented in Supplementary Material 3). Those pdfs provide a complete description of the LFP signal obtained from a given sample and embed all the stochasticity involved in the phenomenon. We suggest that such information is necessary in order to create synthetic LFPs recordings that exhibit all the features of a population and can be applied to a number of pharmacologically and/or genetically treated samples in order to detect subtle but significant differences that might be missed in more traditional analysis methods. This way various hypotheses can be tested faster, while reducing the effort and cost required for biological validation/evaluation.

## Discussion

The developed automated Local Field Potential (LFP) signal analysis pipeline was designed in order to provide reliable, reproducible and high-throughput analysis to experimental neuroscientists and computational/system biologists. Its implementation relies on solid, well established methods from scientific areas such as machine learning, sound/speech engineering and signal processing. Proper adaptation and refinement of these tools to neurophysiological signals allowed us to generate an unbiased methodology to extract massive amount of information from large datasets.

Given that our methodology is data-driven and fully automated, it is by definition both rapid and highly reproducible, so that analysis by different users will produce the same results. These attributes are crucial for computational high throughput methods. In addition, we demonstrate that the developed package is superior to manual analysis in terms of efficiency, as it can detect the smaller events that are close to the noisy baseline of the recording. This is due to the fact that it relies on locally implemented data-driven criteria, which are based on the energy fluctuations of the signal. Hence, it not only overcomes the arbitrariness of imposed thresholds, but it also allows efficient identification of all events that are qualitatively distinct from baseline, irrespective of their size, or the particular conditions of each experiment (e.g. electrode resistance, temperature, flow rate etc.) which could affect the signal-to-noise-ratio of the recording. In this way, it avoids biasing the detection against the smaller events, which could conceal potentially significant biological results.

Furthermore, we also show that, on average, the estimated LFP signal property values agree well with the “ground” truth, as measured by expert users. Accuracy was evaluated by how well the results compare to those obtained by manual analysis by an expert user, which is the traditional and most common way to analyze the data. In this comparison, LFPAnalyzer proved to be highly accurate with a [98% accuracy in event occurrence values] and a 15% ‘overestimation’ in event duration compared to the manual approach. It is noteworthy that this overestimation was highly consistent across data files and we hypothesize that it may be due to the low frequency component that is usually contained in the final segment of the events and is not easily detectable by eye.

In order to evaluate the robustness of our method in terms of biological validation, we compared the established effect of GABA_B_ receptor blockade in the regulation of Up state duration (events validated with complementary intracellular recordings^24^). This evaluation revealed a close match to the literature, verifying the application of our methodology in practice. The additional attributes of brain bursts activity that are automatically quantified with our method, further demonstrate the usefulness of having “easily” extracted information from large LFP datasets that can lead to novel hypotheses for biological evaluation.

Hence, we can conclude that the proposed methodology is a useful and trustworthy tool for analyzing LFP signals. This is particularly important because although LFP recordings are routinely used in the study of network function, their analysis has remained largely ad hoc. This lack of generalizability is at least partly due to the nature of the LFP signal, which is highly variable and noisy, requiring a data based/driven analysis method.

## Methods

Figure 1 Panel 2a demonstrates a typical LFP recording consisting of recurring epochs of persistent activity. The analysis and quantification of these events requires their accurate and unbiased detection and feature extraction, both of which are problematic when manually performed: event detection is extremely time-consuming, while their quantification, which pre-supposes the definition of their onset and offset, is highly subjective. This reality urges for an automated and precise method for event analysis. Furthermore, even data-driven automated approaches can have significant shortcomings if they employ global thresholding schemes. Specifically, global approaches do not account to the *local* properties of the signal. The disadvantage of the globally defined threshold is due to the very nature of the estimation itself, as the threshold level will depend not only on the size of the events, but also on their occurrence. In other words, in recordings with events of similar amplitude, the estimated threshold will be shifted towards higher or lower values depending on whether the recording contains more or fewer events, respectively. Thus, in order to surpass this limitation and be applicable to recordings of different characteristics, a method needs to be adaptive *in time* (please refer to SM5 for a demonstration of the benefits of a local over a global thresholding scheme).

The workflow chart (Fig. 1 Panel 1) highlights the main steps of the proposed methodology, including (a) pre-processing of the LFP signal, (b) creation of the feature signals, (c) estimation of the data-driven threshold, and (d) detection and quantification of the LFP events.

### LFP Signal preprocessing

The input signal is first transformed to analog, i.e. any inherent DC offset due to the acquisition process and the amplifier is subtracted; and the time-series is filtered with a 3rd order Butterworth low-pass filter at 200 Hz, since higher frequencies do not contain LFP related information. The choice of the filter is justified considering the optimal tradeoff between phase shift and minimum signal distortion. An example of the signal before and after the preprocessing step is illustrated in Fig. 1 Panel 2b.

### Feature signal creation

Due to the non-stationary nature of the LFP signal, existing processing methods are not directly applicable. In order to overcome this issue, we divide the preprocessed signal into non-overlapping segments, which can be considered stationary. Each frame is 11 s long, to ensure the inclusion of sufficient baseline, apart from event(s). The choice of 11 s frame length is justified by the empirical knowledge that an event is not expected to be more than 8 s long, and that apart from events, baseline information is required in order to estimate the appropriate threshold. Subsequently, two complementary transformations are applied in order to create two feature signals, which will both be used for optimal event detection since they supply complementary information:

*Hilbert Transform* is a linear operation that takes a signal *u*(*t*) and transforms it to *H*(*u*(*t*)), in the same domain, i.e. time domain^21^. Hilbert Transform is a basic tool in Fourier analysis, and provides a concrete means for realizing the harmonic conjugate of a given function or Fourier series. It is also implemented in the field of signal processing where it is used to derive the analytic representation of a signal *u*(*t*). When *u*(*t*) is narrow-banded, *|H*(*u*(*t*))| can be regarded as a slow-varying envelope of *u*(*t*) while the phase derivative *∂*_*t*_[tan^−*1*^(*y*/*x*)] represents the instantaneous frequency. Thus, Hilbert Transform can be interpreted as a way to represent a narrow-band signal in terms of amplitude and frequency modulation, rendering it well suited for the LFP signal. Additionally, it has been successfully applied for latency analysis in neurophysiological signals^37, 38^. By employing the Hilbert Transform, we create the first feature sequence that corresponds to the signal’s envelope (Fig. 1 Panel 2c).

*Short Time Energy Transform* is a method broadly used in the field of speech processing. It has been proved very effective for detecting silent periods in audio signals and also for discriminating between audio classes^27^. The LFP signal is very similar to speech signal since both are produced from a time varying electrical burst/vocal tract system, with time varying excitation and therefore both are non-stationary in their nature. Short Time Energy is defined as: Let *u*_*i*_(*n*), *n* = *1*, *2*, *…*, *N* the signal samples of the *i*-th segment with length equal to *N*. Then, as shown in Fig. 1 Panel 2d, the energy is calculated for each frame as:

1 $$E(i)=\frac{1}{N}{\sum }_{n=1}^{N}|{u}_{i}(n){|}^{2}$$

The two feature signals are used in order to estimate two thresholds (one for each signal; Fig. 1 Panel 2e,f) and their combination will exhibit the final detected events. Both are needed since each one on its own has potential drawbacks: the Hilbert transform is not sensitive to the low amplitude periods of signal leading to the erroneous exclusion of parts of events, while short time energy is very sensitive to small “pauses” within an event leading to over-segmentation.

### Dynamic Threshold Estimation

For estimating the threshold that will be used to separate event from non-event signal segments, we employ the Gaussian Mixture Model (GMM), a data driven approach borrowed from machine learning^28^. The thresholds, one for each feature signal, are dynamically defined by the nature of the input data. Here GMMs have been modified in order to be used in an unsupervised manner^39^, i.e. requiring no pre-training. The final number of classes present in the data is defined by the algorithm itself, using an information criterion discussed below. Mixture models are a very flexible and powerful tool in modern pattern recognition. The presented application consists in fitting Gaussian Mixtures to the observations, i.e. the voltage traces, without a priori knowledge of the optimal number of components needed.

For each segment of the transformed signal (feature signal), a histogram of the voltage values is created. The maximum number of classes is defined as two, which is the “natural” number, following the rationale that the high (absolute) voltage values are part of LFP events, and low values are part of the baseline signal.

The categorization into ‘event’ or ‘baseline’ is performed based on the histogram of object pixel intensities using 1-D GMM and a modified Expectation–Maximization (EM) algorithm^39, 40^. The signal segments can have two distinct patterns: a segment will either contain both baseline and event signal (partial or whole event), or only baseline. In the first case the histogram should exhibit two peaks (a model of two Gaussians explains the data distribution), whereas in the second case there would be only one peak (one Gaussian function suffices to account for the data distribution). Hence, the developed algorithm should have the ability to automatically define the number of Gaussians in either case and set the threshold accordingly. In order to do that, we employ the Minimum Message Length (MML) criterion for automatic model selection. The MML criterion^41^ is incorporated into the EM in order to determine the mixture model with the optimal number of components (either 1 or 2 in our case) that best fits the voltage histogram. In cases where two classes are adequate to represent the data, then the threshold is determined automatically under the Bayes rule^41^. If one class is a better representative for the data, then no threshold is determined and the signal is considered as baseline. This is an important novel feature compared with previous studies that use a fixed 2 component GM^28^, as it provides the required flexibility for accurate threshold estimation in recordings with different properties, such as event occurrence and stability of baseline.

At the end of this procedure, we end up with two adaptive thresholds for each recorded trace, one for each feature signal (Hilbert and Short Time Energy transformed signals), indicated as red lines in Fig. 1 Panel 2e,f. When the threshold is above the maximum of the signal then the underlying period is by default considered as event free.

### Mask derivation and event detection

Each threshold is applied to the corresponding feature signal and a mask is created. The mask consists of a signal of segments of zeros and ones (Fig. 1 Panel 2i; red line which is scaled to the maximum amplitude value for visualization purposes), where the periods of ones define the presence of a prospective event (i.e. above threshold), while periods of zeros indicate the baseline (i.e. below threshold). Then, the two masks are combined using a logical OR operator. In order to avoid the false detection of mechanical and/or electric noise artifacts with fluctuations of size and duration similar to the spontaneous brain activity, an additional post-processing step is applied in order to exclude small artifacts: events with standard deviation (SD) smaller than the standard deviation of the whole signal are excluded. The rationale behind this step is that LFP events will exhibit higher SD values compared to the overall signal which contains large segments of baseline with small amplitude variability. Finally, simultaneously to the LFP burst detection, a segment of stable baseline is also automatically detected, as optimal baseline representative. This is achieved by selecting the longest event-free period so as not to include periods with voltage fluctuations.

### Additional features of the analysis workflow

After detection of the LFP events, a number of their properties are calculated and stored in Excel and MATLAB data files (please refer to https://zenodo.org/record/59254 for an output example). These include: (a) onset, (b) offset, (c) event duration, (d) inter-event interval (time interval between the offset of one event and the onset of the next), (e) time point for maximum peak amplitude, (f) maximum peak amplitude, (g) time point for minimum peak amplitude, (h) minimum peak amplitude, and (i) rectified area (the sum of absolute voltage values of the events). Furthermore, the power spectrum of each LFP event is calculated for several frequency ranges, including the continuous power spectrum, as well as the delta (1–4 Hz), theta (4–8 Hz), alpha (8–12 Hz), beta (12–30 Hz), and gamma (30–100 Hz and 30–120 Hz) bandwidths, using the conventional Fourier analysis and multi-taper method^42^. Additionally, two types of the calculated power spectra are also computed: the power spectra of the LFP bursts before and after the removal of the baseline power spectrum. For the above power estimation, we calculate three additional types of spectra for each event: absolute, normalized to the total power of the event and normalized to the maximum value of power coefficient. The normalization procedure allows the direct comparison of the % differences, since LFP bursts within or between recordings can differ significantly in both amplitude and duration, and consequently in their absolute power values.

The developed software package is a full suite that provides the user a large variety of useful properties. At the beginning of the pipeline the user is asked to insert information on the experiment performed, i.e. animal genotype, age and sex, its pharmacological manipulations etc. All this information is registered in a separate excel sheet for user’s future reference and downstream statistical processing of the results.

### Implementation and performance

The signal processing was implemented in MATLAB 2012a, where known methods have been adapted and tailored to the present application. The final GUI (Graphical User Interface) is platform-independent and currently is compiled for Win x64 environment. It does not require the MATLAB environment to be installed, but it requires the MATLAB Component Runtime installer. The computational time for the whole analysis for each single channel, with the time for user to pass the results from a rough quality check (i.e. remove artifacts), is less than 2 minutes. The largest portion of this time accounts to the final result saving into excel files (.xlsx).

### Software and data availability

The GUI is available at https://zenodo.org/record/59254, along with an input file (contains 4 channels, i.e. 4 recordings). This file contains the raw recordings of one from control sample and holds 4 simultaneous recordings. As stated earlier, the GUI does not require installation of MATLAB suite and the only prerequisites are: (1) a windows x64 operating system, and (2) an installed MATLAB Runtime compiler. MATLAB runtime is freely available at http://www.mathworks.com/products/compiler/mcr/ (version R2012a (7.17)).

### Animals

C57Bl/6J mice were bred in the animal facility of the Center for Experimental Surgery of the Biomedical Research Foundation of the Academy of Athens. The facility is registered as a breeding and experimental facility according to the Presidential Decree of the Greek Democracy 160/91, which harmonizes the Greek national legislation with the European Council Directive 86/609/EEC on the protection of animals used for experimental and other scientific purposes (reference number 2834/08-05-2013). Mice were weaned at postnatal day 21, housed in groups of 5–9, in 267 × 483 × 203 mm cages supplied with bedding material and kept at a 12-12 dark-light schedule. Food was provided ad libitum.

### Brain slice preparation

Coronal brain slices (400 μm) from the primary somatosensory (S1) cortex were prepared from adult (3–9 months old) and aged (20–27 months old) male mice, as previously described^29, 30^. After the animal was sacrificed the brain was removed and placed in an oxygenated (95% O_2_–5% CO2) ice-cold dissection solution containing, in mM: KCl 2.14; NaH_2_PO_4_.H_2_O 1.47; NaHCO_3_ 27.0; MgSO_4_ 2.2; D-Glucose 10.0; Sucrose 200; and CaCl_2_.2H_2_O 2.0; osmolarity (mean ± sd): 298 ± 5 mOsm, pH: 7.4. Slices were cut using a vibratome (VT 1000 S, Leica), placed in a holding chamber with artificial cerebrospinal fluid (aCSF) containing, in mM: NaCl 126; KCl 3.53; NaH_2_PO_4_.H2O 1.25; NaHCO_3_ 26.0; MgSO_4_ 1.0; D-Glucose10.0 and CaCl_2_.2H_2_O 2.0 (osmolarity (mean ± sd): 317 ± 4 mOsm, pH: 7.4), and left to recover at room temperature (RT: 24–26 °C) for at least one hour before use.

### ***In vitro*** electrophysiology

Slices were transferred to a submerged chamber (Luigs and Neumann), where they were constantly perfused at high flow rates (10–15 ml/min) to ensure optimal oxygenation of the cortical tissue^43, 44^. Recordings were performed in “*in vivo* like” aCSF (composition as above but with 1 mM CaCl_2_), since this ionic solution is thought to better mimic cerebrospinal fluid *in vivo* ^44, 45^. Recordings were performed at RT, after at least 30 min of incubation in 1 mM [CaCl_2_] aCSF. Spontaneous network activity was assessed by means of local field potential (LFP) recordings (sampled at 5 or 10 kHz, band-passed filtered at 1–3000 Hz) obtained from cortical layers II/III using low impedance (∼0.5 MΩ) glass pipettes filled with aCSF. Signals were acquired and amplified (MultiClamp 700B, Axon Instruments), digitized (Instrutech, ITC-18) and viewed on-line with appropriate software (Axograph X, version 1.3.5).

### Pharmacology

Recordings were also obtained in the presence of the GABA_B_-receptor- antagonist, CGP55845 (1 μM, Tocris). After recording baseline activity in the absence of any drugs, CGP55845 was added to the perfusion medium and the LFP signal was monitored for 30 minutes.

### Electrophysiological data summary

Coronal brain slices (400 μm) from the primary somatosensory cortex were prepared from adult (3–9 months old) and aged (20–27 months old) male mice and spontaneous network activity was assessed by means of local field potential (LFP) recordings, as previously described^34, 35^. Recordings were also obtained in the presence of the GABA_B_-receptor-antagonist, CGP55845^36^ (1 μM, Tocris). In total, the dataset consisted of 36 paired recordings (6 animals) before and after the application of the GABA_B_ antagonist.

### Statistical Analysis and Linear Regression

Statistical analysis (t-test) was performed in Matlab environment using t-test function from Statistics Toolbox. For the values resulted for the Accuracy and Reproducibility subsections of the Results Section, we applied linear regression using the linear model $f(x)={a}^{\ast }x+b$, where *α* is the gradient of the fitted line and *b* is the bias, i.e. the estimated difference between the two methods (see also Supplementary Material 4). Linear Regression was also performed in Matlab using the Curve Fitting Toolbox (cftool). Data were scaled and centered, and LAR^46^ robust method was used. Least absolute residuals (LAR) method finds a curve that minimizes the absolute difference of the residuals, rather than the squared differences. Therefore, extreme values have a lesser influence on the fit.

## Electronic supplementary material

Supplementary Info
