# Supplementary material for: High-Throughput Analysis of in-vitro LFP Electrophysiological Signals: A validated workflow/software package
Source: Sci Rep. 2017 Jun 8;7:3055. doi: 10.1038/s41598-017-03269-9 (PMC5465098; doi:10.1038/s41598-017-03269-9)
Supplement: Supplementary file 1 — Supplementary Info [file 41598_2017_3269_MOESM1_ESM.pdf]

High-Throughput Analysis of in-vitro LFP Electrophysiological Signals: A validated workflow/software package

Tsakanikas P., Sigalas C., Rigas P., Skaliora I.
Biomedical Research Foundation, Academy of Athens, Center of Basic Research
{tsakanikas, csigalas, prigas, iskaliora}@bioacademy.gr

Supplementary Material 1

Regression Analysis of Duration, Onset and Offset values from the same dataset; analyzed by different users.

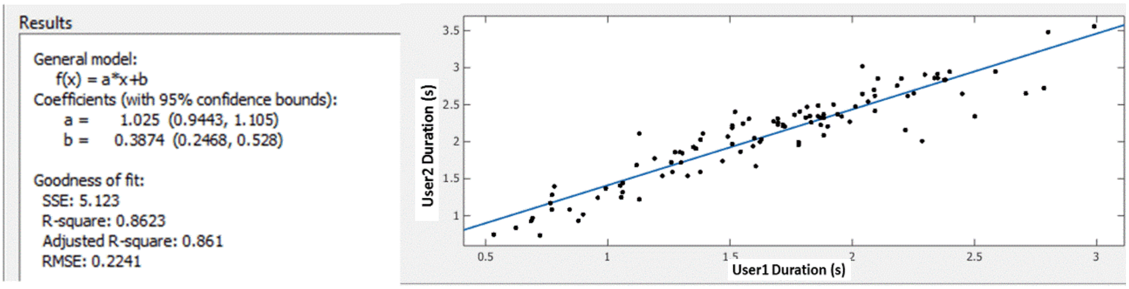

Fig. SM1.1: Regression analysis on duration.

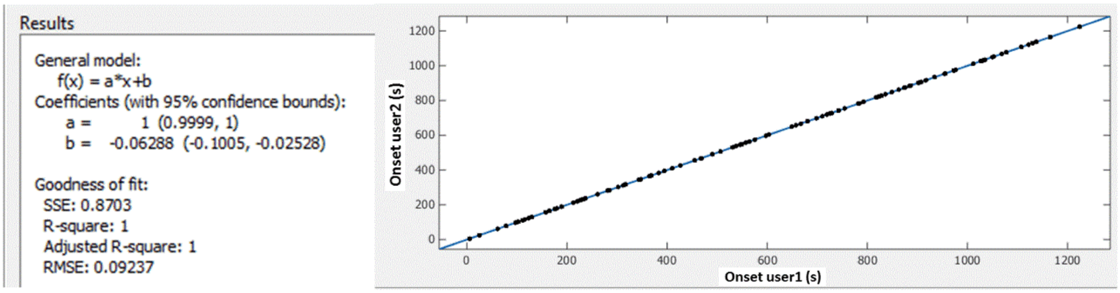

Fig. SM1.2: Regression analysis on Onset.

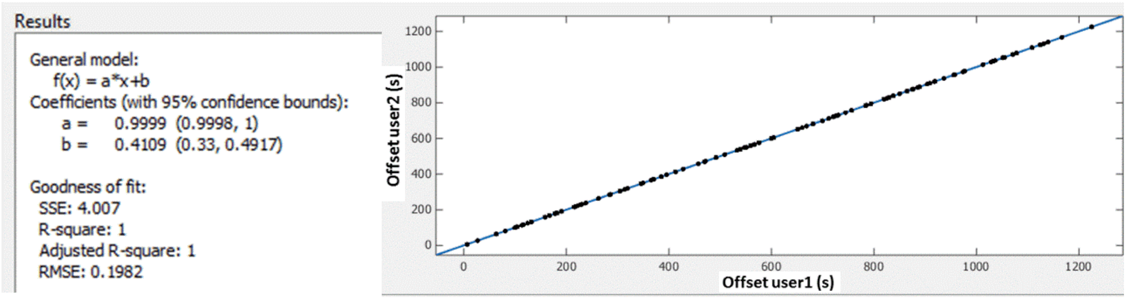

Fig. SM1.3: Regression analysis on Offset.

Supplementary Material 2

Examination of statistical significance of spectral information of shared (LFPAnalyzer and user defined) events among the several frequency bands.

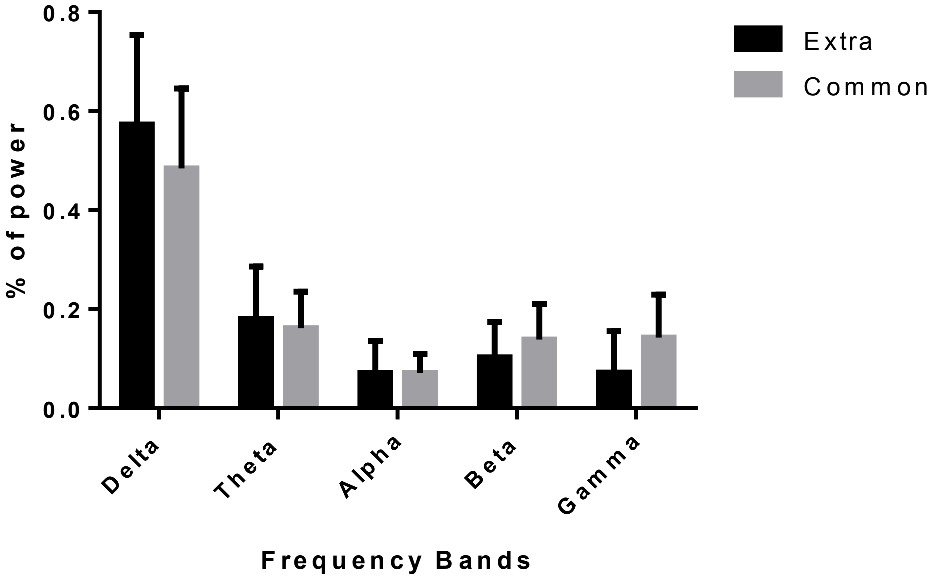

Fig. SM2.1: Percentages over total power of each frequency band power and for common and extra detected events. Bars are the average percentages while errors bars the standard deviations.

Table SM2.1: p-values computed for each frequency band with unpaired t-test.

	Delta	Theta	Alpha	Beta	Gamma
**p**	0.392	0.733	0.992	0.400	0.180

Supplementary Material 3

Pdfs are defined as: $p(x|\lambda)=\sum_{i=1}^{M} w_i g(x|\mu_i,\Sigma_i)$, where x is the 1-dimensional observations of each parameter, w_i, i=1,..,M the mixture weights with $\sum_{i=1}^{M} w_i = 1$, *g* is the Gaussian function, μ and Σ the mean and covariance and λ the full set of parameters $\lambda=\{w_i,\mu_i,\Sigma_i\}$. We used a two component GMM, thus *M*=2, and the resulting parameters are shown in Table SM3.1.

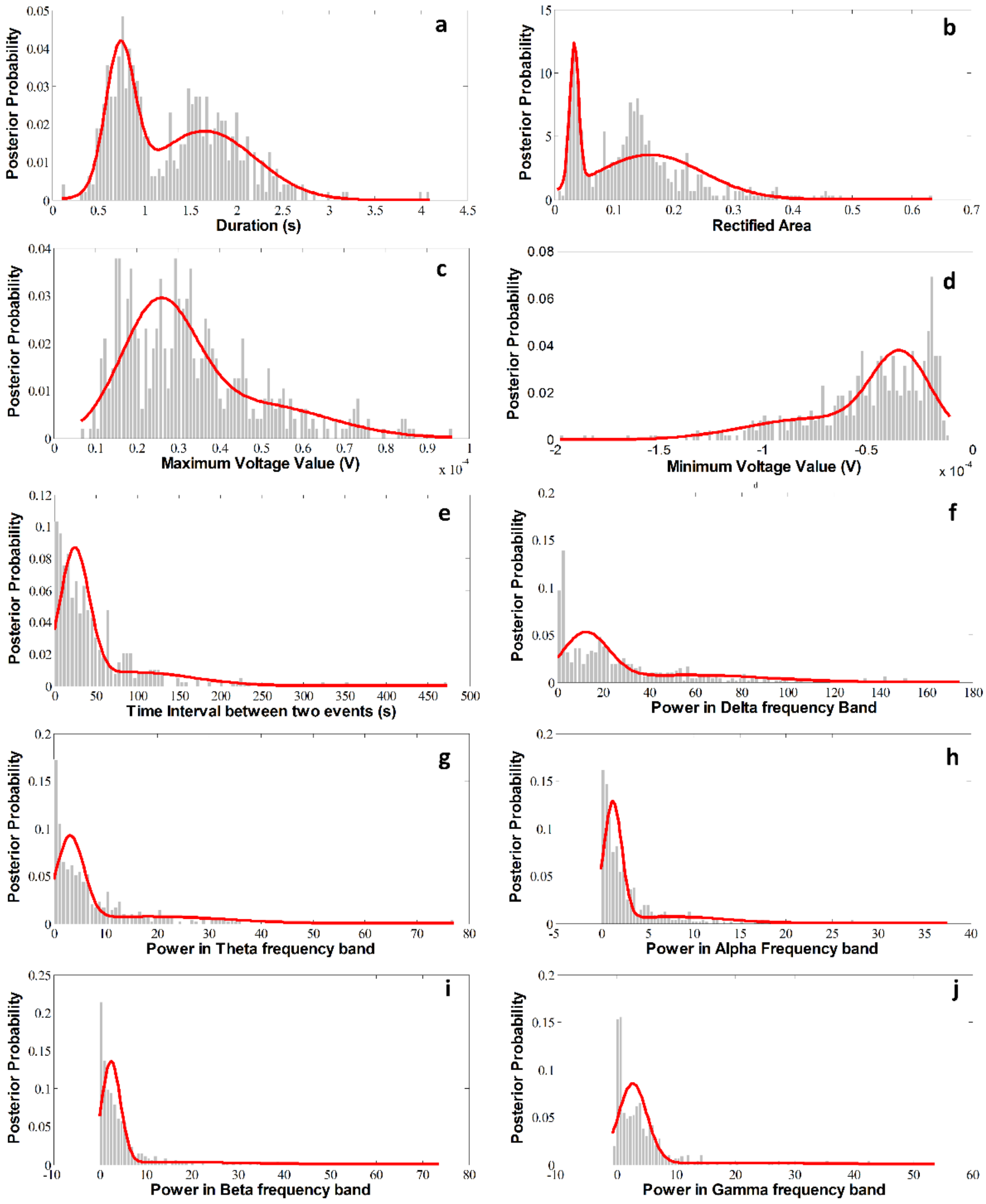

Fig. SM3.1: Probability density estimation via GMMs with 2 components for: (a) Duration, (b) Rectified area, (c) Maximum voltage values, (d) Minimum voltage values, (e) Time interval between two consecutive events, (f) Power in Delta frequency band, (g) Power in Theta frequency band, (h) Power in Alpha frequency band, (i) Power in Beta frequency band, (j) Power in Gamma frequency band.

Table SM3.1: Estimation of the parameters for the probability density estimation via GMMs with 2 components for: (a) Duration, (b) Rectified area, (c) Maximum voltage values, (d) Minimum voltage values, (e) Time interval between two consecutive events, (f) Power in Delta frequency band, (g) Power in Theta frequency band, (h) Power in Alpha frequency band, (i) Power in Beta frequency band, (j) Power in Gamma frequency band. μ is the mean value, Σ is the covariance and w the weight for each Gaussian component correspondingly.

	M	Σ	W
**Duration**	0.74<br>1.65	0.02<br>0.31	0.37<br>0.63
**Rectified Area**	0.11<br>0.28	$3.82\ 10^{-3}$<br>$12.97\ 10^{-3}$	0.84<br>0.16
**Maximum Voltage**	$4.84\ 10^{-5}$<br>$2.52\ 10^{-5}$	$2.97\ 10^{-10}$<br>$8.05\ 10^{-11}$	0.34<br>0.66
**Minimum Voltage**	$-7.69\ 10^{-5}$<br>$-3.44\ 10^{-5}$	$1.06\ 10^{-9}$<br>$2.03\ 10^{-10}$	0.37<br>0.63
**Time Interval**	24.45<br>97.36	313.97<br>4815.77	0.71<br>0.29
**Delta Power**	56.45<br>12.30	1183.53<br>101.27	0.34<br>0.66
**Theta Power**	18.11<br>3.20	212.57<br>6.95	0.32<br>0.68
**Alpha Power**	7.52<br>1.22	37.64<br>0.95	0.26<br>0.74
**Beta Power**	18.50<br>2.46	245.74<br>4.21	0.16<br>0.84
**Gamma Power**	2.74<br>20.88	5.97<br>200.96	0.88<br>0.12

Supplementary Material 4

Regression Analysis of differences in Duration, Onset and Offset values between LFPAnalyzer and Manual estimation.

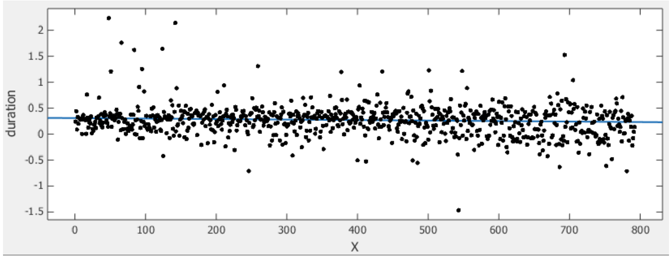

Linear model Poly1:
f(x) = a*x+b
where x is normalized by mean 396.5 and std 228.8
Coefficients (with 95% confidence bounds):
a = -0.02178 (-0.02414, -0.01942)
b = 0.2708 (0.2685, 0.2732)

Goodness of fit:
SSE: 0.9002
R-square: 0.9875
Adjusted R-square: 0.9875
RMSE: 0.03376

Fig. SM4.1: Regression analysis on duration differences.

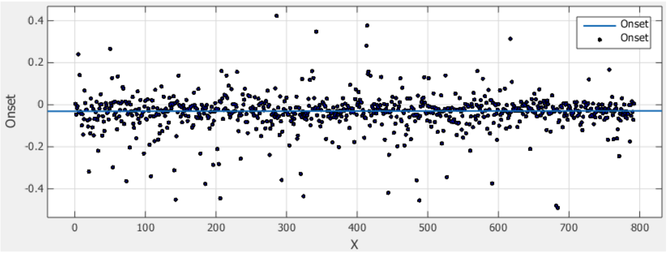

Linear model Poly1:
f(x) = a*x+b
where x is normalized by mean 396.5 and std 228.8
Coefficients (with 95% confidence bounds):
a = 0.0005446 (-0.0006596, 0.001749)
b = -0.03015 (-0.03136, -0.02895)

Goodness of fit:
SSE: 0.2352
R-square: 0.9622
Adjusted R-square: 0.9622
RMSE: 0.01725

Fig. SM4.2: Regression analysis on Onset differences.

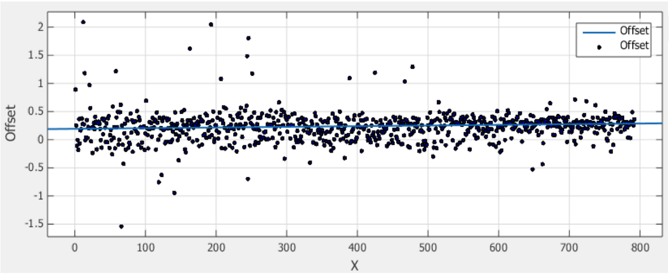

Linear model Poly1:
f(x) = a*x+b
where x is normalized by mean 396.5 and std 228.8
Coefficients (with 95% confidence bounds):
a = 0.02688 (0.02209, 0.03167)
b = 0.2397 (0.235, 0.2445)

Goodness of fit:
SSE: 3.715
R-square: 0.9338
Adjusted R-square: 0.9337
RMSE: 0.06857

Fig. SM4.3: Regression analysis on Offset differences.

Supplementary Material 5

Quantitative demonstration of the benefits of the local vs. the global thresholding approach in (a) the reliable detection of all LFP events, and (b) the accurate estimation of the onset and offset of each event.

We first compare the reliability and accuracy of event detection using a local/adaptive thresholding scheme and a global thresholding scheme on the same dataset. As can be seen in fig SM5.1, the calculated threshold value when the entire trace is used (i.e. global thresholding, as in the Mukovski et al 2007 paper) is higher than when a local thresholding approach is used. This is especially prominent in cases of high occurrence of LFP events (as this example) and less prominent in cases of low occurrence (Fig. SM5.2 b). In such cases, many small events fail to be detected, as evident when comparing panels c (globally estimated threshold) and d (locally estimated threshold). In addition, the onset and offset of the detected events is more accurate in the case of local thresholding, since the estimation of the threshold values takes into account the local properties of the signal. Panel e indicates the estimated onset/offset based on global thresholding, as in the Mukovski et al. 2007 study), whereas panel f shows the estimated onset/offset based on adaptive local thresholding, as in the present study. It is clear that the global thresholding approach leads to tighter event limits (i.e. onsets and offsets), where the low frequency component at the end of each event is excluded and as presented in Fig. SM5.2 f, not in a systematic way. In addition, longer events that may include a brief period of lower amplitude signal are being fragmented into separate events of shorter duration (as in the event on the left in panel e).

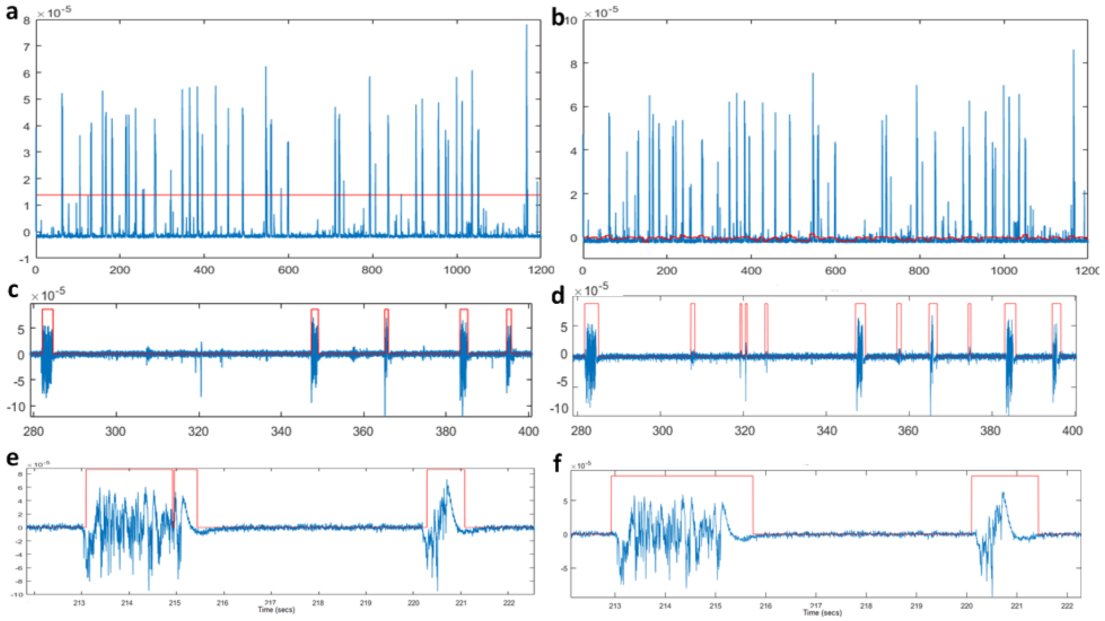

Fig. SM5.1. *Application of globally vs locally derived thresholds for event detection in the same dataset. a) Transformed (Hilbert Transform) recorded signal with the estimated threshold (red line) using global estimator (i.e. the entire recorded trace). b) Transformed (Hilbert Transform) recorded signal with the estimated threshold (red line) using local estimator (as described in the present manuscript). c-d) detected events for the global and local threshold, respectively.*

e-f) higher magnification of the detected events by the global (e) and the local approach (f), indicating the onset and offset estimation (red lines).

In order to further demonstrate the benefits of our approach, we present the results obtained with the global thresholding scheme on two recordings with different occurrence rates (Fig. SM5.2). As can be seen, the threshold value obtained with the global thesholding approach (as used in the Mukovski et. al. 2007 paper) for the high occurrence dataset (panel a) leads to the detection of events with average amplitudes around 5×10^{-5} V, while excluding almost every event with amplitudes around 2×10^{-5} V (panel c). At the same time, the events detected in a different dataset with lower event occurrence (panel b) have amplitude values at around 2×10^{-5} V (panel d). This implies that if one wants to compare the network activity in those two recordings they would have erroneously *excluded* events from the high occurrence recording that are similar (at least in terms of amplitude) to events of the low occurrence recording. Furthermore, the duration of events detected in the lower occurrence recording is *overestimated* compared to the duration of detected events in the high occurrence recording (compare panels e and f).

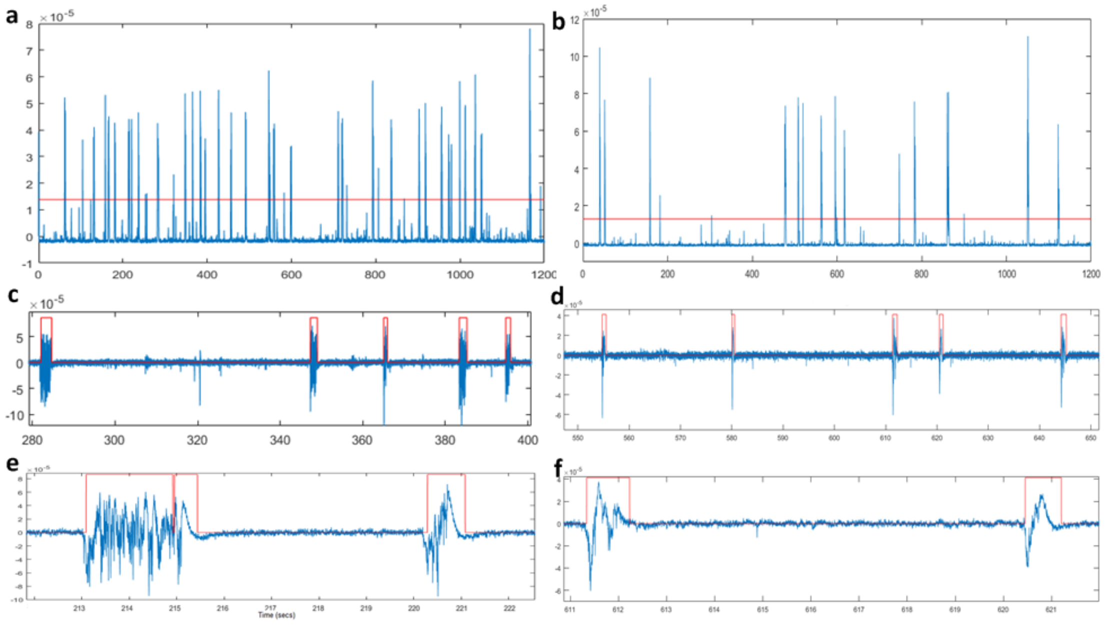

Fig. SM5.2. *Application of globally derived thresholds for event detection in two datasets with different event occurrence. a-b) Transformed (Hilbert Transform) signal with the estimated threshold (red line) using global estimator. c-d) detected events for the different signals with global threshold. e-f) higher magnification of the detected events by the global approach, indicating the onset and offset estimation (red lines).*

It is clear from the above analysis that the application of local threshold estimator for event detection is more robust compared to the global one, as it leads to (i) the detection of more events, (ii) more reliable event detection across different recordings (see Fig. SM5.1 e,f), (iii) more accurate estimation of the absolute event duration, and (iv) more reproducible estimation of event duration among recordings of different signal-to-noise ratios.
